# Anæsthesia Cured by the Application of Metal

**Published:** 1879-02

**Authors:** 


Anæsthesia Cured by the Application of Metal.—After the announcement of Burcq, that the anæsthesia of hysterical persons could be cured by the application of metals, had been confirmed by the experiments of Charcot, Adamkiewicz undertook to discover the ground of this peculiar phenomenon. The statement of the French author, that every artificial production of sensibility in persons laterally anæsthetic was united with a diminution of sensibility at a corresponding spot on the sound side, interested him. He had found that certain nervous processes, as also the secretion of sweat, invariably took place in men bilaterally. The statement of the French author, that the skin-spots made sensitive by the metal became red, and bled when pricked, led the reporter to believe that the mysterious effect of the metal was due entirely to the mechanical irritation. In order to prove this view, Adamkiewicz applied mustard plasters instead of the metal. The result was a positive one. Under the influence of the mustard the anæsthesia disappeared, sometimes completely. In one case for a few days, in others permanently. So also the above mentioned symptoms, described by the French as "transport de la sensibilite," the reporter (Adamkiewicz) could produce with his mustard plasters.—*Med. Neuigk.* No. 2, 1879.

